# A Rare Case of Staphylococcus lugdunensis Causing a Pseudoaneurysm of the Aorta

**DOI:** 10.7759/cureus.24530

**Published:** 2022-04-27

**Authors:** Patrick Huynh, Ashwin Mathew, Ajinkya Buradkar, Pooja Kharbanda, Rahul Rauniyar

**Affiliations:** 1 Internal Medicine, Northside Hospital, Lawrenceville, USA; 2 Internal Medicine, The University of Edinburgh, Edinburgh, GBR; 3 Internal Medicine, The Wright Center for Graduate Medical Education, Scranton, USA

**Keywords:** staphylococcus lugdunensis, staphylococcus, pseudoaneurysm

## Abstract

*Staphylococcus lugdunensis* (SL) is a well-known skin commensal. It is coagulase-negative bacteria that has often been labeled as a contaminant. While coagulase-negative bacteria are not as virulent as *Staphylococcus* aureus, there has been an increasing trend for this organism to be associated with complications commonly known to occur with its more aggressive counterpart. We report a case of *Staphylococcus lugdunensis* causing infective endocarditis and pseudoaneurysm of the aorta.

## Introduction

*Staphylococcus lugdunensis* is a coagulase-negative staphylococcus and well-documented organism, commonly found on the skin. It has also been a known culprit in skin and soft tissue infections. However, it can rarely cause fatal bacterial endocarditis. In rare cases, it can cause pseudoaneurysms of the aorta. We present a case of a patient with a large pseudoaneurysm of the ascending aorta caused by *Staphylococcus lugdunensis*.

## Case presentation

We present a case of a 52-year-old Hispanic male with a history of bioprosthetic aortic valve replacement four years prior and tetralogy of Fallot (during childhood) presented with chest pain and abdominal pain for the past three weeks. The chest pain was substernal in location. The patient described the pain as 7/10 intensity and radiated to the epigastric region. He did not have associated diaphoresis, palpitations, or nausea. The patient was recently treated for aortic valve endocarditis with long-term intravenous antibiotic therapy lasting six weeks. The patient was admitted with a one-month history of fever, chills, and left upper quadrant discomfort. Upon presentation, he was febrile with laboratory values that were significant for leukocytosis and mild troponemia. The patient was admitted with a working diagnosis of sepsis of unknown etiology. Blood cultures were drawn, and the patient was started on broad-spectrum antibiotics.

**Figure 1:**
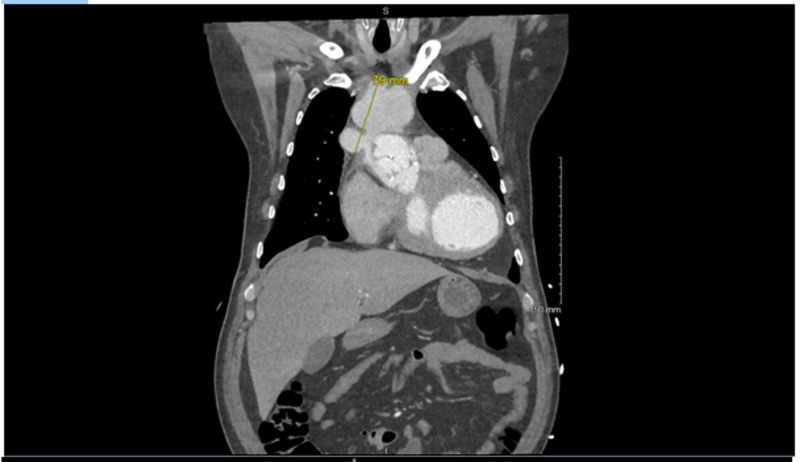
Coronal view of CT scan demonstrating the pseudoaneurysm of the aorta; yellow line demarcates the pseudoaneurysm with exact dimensions.

**Figure 2:**
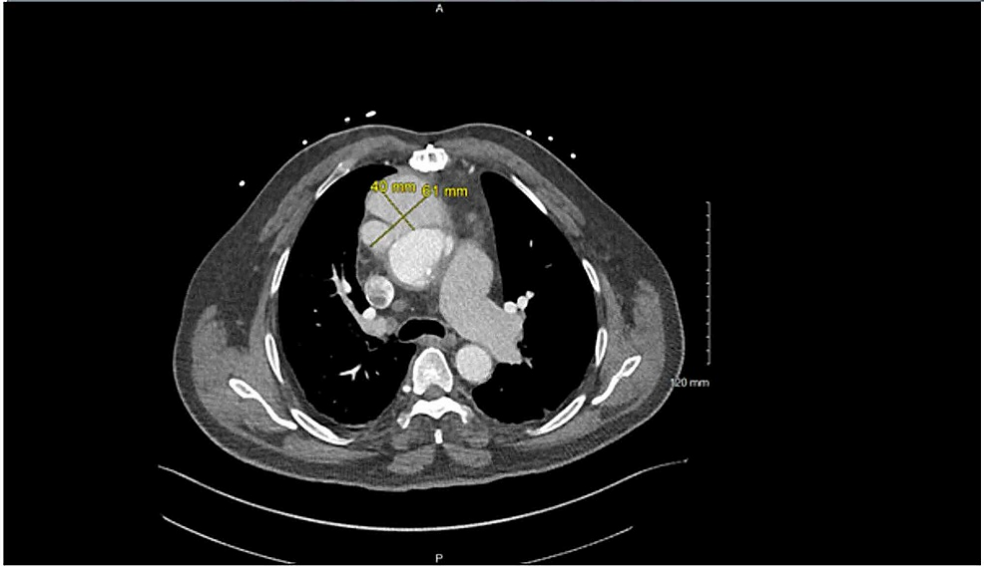
Trans-axial view of CT scan demonstrating the pseudoaneurysm of the aorta; yellow line demarcates the pseudoaneurysm with exact dimensions.

Echocardiogram was suggestive of a vegetation involving the bioprosthetic aortic valve. Computed tomography of the chest revealed signs of a pseudoaneurysm involving the ascending aorta (Figures 1, 2). Blood cultures eventually grew coagulase-negative staphylococci. The patient was ultimately cleared for surgery and prepped for pseudoaneurysm repair with aortic bioprosthetic valve replacement. However, while undergoing the repair, the patient experienced cardiac arrest during the procedure. Thus, further intervention was canceled. Due to the complicated intra-operative course, no intra-operative pictures were taken and no fragments of tissue were able to be cultured for growth. The patient was sent to the ICU and subsequently switched to hospice care after the cardiac arrest. No further intervention was pursued.

## Discussion

We present a case of a 52-year-old Hispanic male with a medical history of bioprosthetic aortic valve replacement and tetralogy of Fallot (during childhood) corrected by modified Bentall procedure who is diagnosed with *Staphylococcus lugdunensis* infective endocarditis and pseudoaneurysm formation of the ascending aorta. We believe this to be one of the first few cases published of a pseudoaneurysm forming in the ascending aorta secondary to *Staphylococcus lugdunensis *(SL) endocarditis.

SL is coagulase-negative staphylococci and is a common commensal found on the skin, but predominantly over the perineal area [1]. It is distinguished from other coagulase-negative staphylococci such as *Staphylococcus epidermidis* by the presence of pyrrolidonyl arylamidase and ornithine decarboxylase activity on biochemical testing.

It is well-known that SL not only causes skin and soft tissue infections but is also implicated as a rare cause of bacterial endocarditis. A study by Herchline and Ayers showed that SL is only found to be a contaminant in 15.4% of isolates and should generally be considered a true pathogen, especially in deep-seated infections [2].

SL is well known for its propensity towards an aggressive clinical course and is said to behave similarly to *Staphylococcus aureus*. Although this rare microbe has a predilection to affect native valves more often, prosthetic valve involvement has been linked with a more sinister outcome, with higher rates of complications such as heart failure, periannular abscess, embolization, shock, and death [3]. Mortality rates are found to be around 38.8-70% with higher rates being found in prosthetic valve involvement as compared to native valves [3,4].

The formation of mycotic aneurysms (pseudoaneurysms) secondary to SL is found to be an extremely rare occurrence with most of the data being available only through case reports. Pseudoaneurysms in these cases were found to involve the intracranial vessels such as the middle cerebral artery [5] and also extracranial sites, such as the left ventricle [6], subvalvular portion of aorta [7], ascending aorta [8], superior mesenteric artery [9,10], and tibial arteries [5]. The mechanism underpinning the aggressive nature of SL is largely unknown. However, it is postulated that certain adherence proteins such as von Willebrand factor (vWF) and fibrinogen-binding proteins may aid in the adherence of the bacterium to tissue and artificial surfaces [11]. The dislodgement of septic emboli may also cause seeding of the bacteria in distant sites, thus explaining the various locations of mycotic aneurysms mentioned above. Besides that, a unique feature of SL is the formation of biofilm which thus contributes to impaired host response towards the bacterium and also reduced penetration of antibiotics [11].

It is vitally important to recognize SL endocarditis as early as possible in view of its high mortality. A case review by Pada et al. recommends the use of 16S ribosomal DNA sequencing for the early diagnosis of SL bacterial endocarditis [12]. Although SL is associated with susceptibility to a large number of antibiotics, a purely medical approach has been shown to confer higher mortality [4]. Therefore, it is advisable to consider an early surgical intervention in these cases.

## Conclusions

This case highlights the importance of considering broad differentials, especially in the patients with extensive past medical history of structural and valvular heart disease, as these patients often have higher mortality rates in the setting of common skin commensals such as SL. It is vital to keep *Staphylococcus lugdunensis* in mind as a differential organism for bacterial endocarditis, especially in the presence of a pseudoaneurysm. When treating such a patient, aggressive initiation of antimicrobial therapy early in the disease course may help mitigate progression of this infection and subsequently formation of pseudoaneurysm. However, most patient often require a dual approach of medical and surgical intervention to appropriately manage these patients and improve outcomes.
